# Cholecystectomy of an Intrahepatic Gallbladder in an Ectopic Pelvic Liver: A Case Report and Review of the Literature

**DOI:** 10.1155/2017/3568768

**Published:** 2017-10-31

**Authors:** Rachel Mathis, Joshua Stodghill, Timothy Shaver, George Younan

**Affiliations:** ^1^Department of Surgery, Inova Fairfax Hospital, Fairfax, VA, USA; ^2^Virginia Surgery Associates, Fairfax, VA, USA

## Abstract

**Introduction:**

Ectopic pelvic liver is an exceedingly rare condition usually resulting after repair of congenital abdominal wall defects. Intrahepatic gallbladder is another rare condition predisposing patients to cholelithiasis and its sequelae. We describe a cholecystectomy in a patient with an intrahepatic gallbladder in a pelvic ectopic liver.

**Presentation of Case:**

A 33-year-old woman with a history of omphalocele repair as an infant presented with signs and symptoms of symptomatic cholelithiasis and chronic cholecystitis, however, in an unusual location. After extensive workup and symptomatic treatment, cholecystectomy was recommended and performed via laparotomy and hepatotomy using microwave technology for parenchymal hepatic transection.

**Discussion:**

Given the rare combination of an intrahepatic gallbladder and an ectopic pelvic liver, advanced surgical techniques must be employed for cholecystectomies, in addition to involvement of hepatobiliary experienced surgeons due to the distortion of the biliary and hepatic vascular anatomy.

**Conclusion:**

Cholecystectomy by experienced hepatobiliary surgeons is a safe and effective treatment for cholecystitis in patients with intrahepatic gallbladders in ectopic pelvic livers.

## 1. Introduction

Ectopic or wandering liver is exceedingly rare outside of the pediatric population and is generally confined to colonic interposition into the right upper quadrant or epigastric region [1–3]. Few reports exist in the literature about a pelvic location of the liver in adults [4, 5]. Intrahepatic gallbladder—one that is partially or completely surrounded by the liver parenchyma—is slightly more common, though also rare [6]. An intrahepatic gallbladder often exhibits impaired function, which may lead to stasis and gallstone formation [7]. Thus, patients with intrahepatic gallbladders are more prone to complications of cholelithiasis, such as cholecystitis. To the knowledge of the authors of this paper, there are only two prior case reports of intrahepatic gallbladders in pelvic livers; both associated with previous omphalocele repair in infancy [8]. Herein, we describe a case of successful surgical treatment of chronic cholecystitis in a woman with a history of omphalocele repair as an infant with an intrahepatic gallbladder in an ectopic pelvic liver.

## 2. Case Presentation

A 33-year-old African American woman with a history of omphalocele repair as an infant presented to the general surgery clinic with left upper and lower quadrant abdominal pain that radiates to the back, nausea related to fatty food ingestion, bloating, diarrhea, and subjective fevers. She had undergone an extensive workup with her gastroenterologist and was found to have cholelithiasis. Her past medical history included gastroesophageal reflux disease, anxiety, depression, and kidney stones. Past surgical history was significant for repair of an omphalocele shortly after birth. Her social history was negative for smoking or alcohol use.

**Figure 1:**
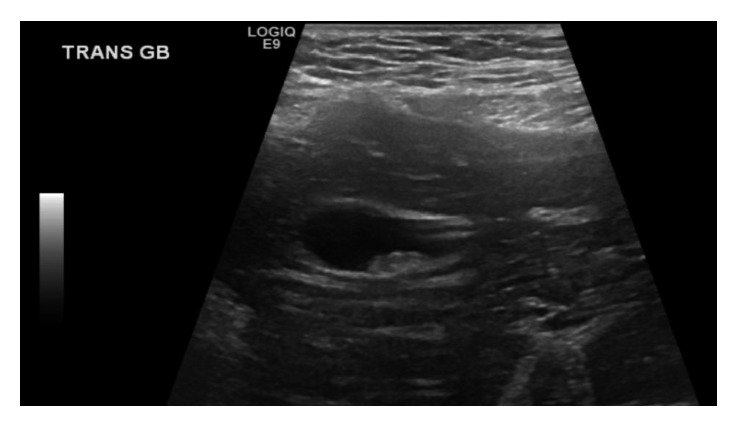
Transabdominal ultrasound demonstrated sludge and stones in a contracted gallbladder.

**Figure 2:**
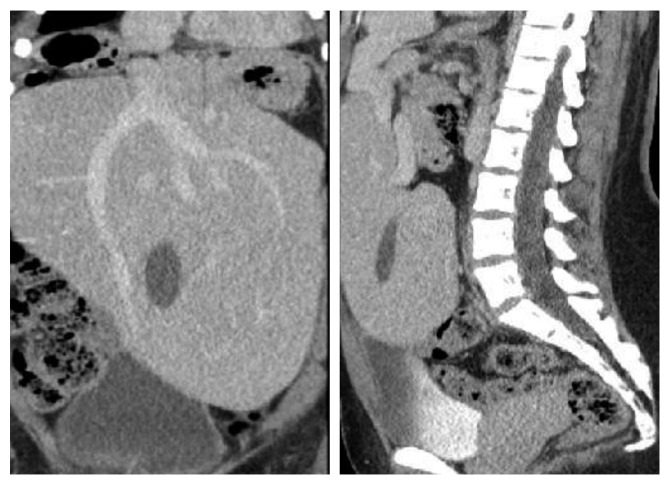
Coronal and sagittal computed tomography pictures showing the pelvic location of the liver and the intrahepatic location of the gallbladder.

**Figure 3:**
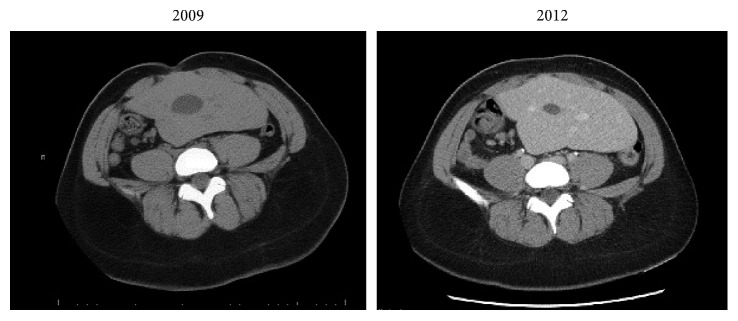
Axial computed tomography pictures showing contracture of the gallbladder from 2009 to 2012.

**Figure 4:**
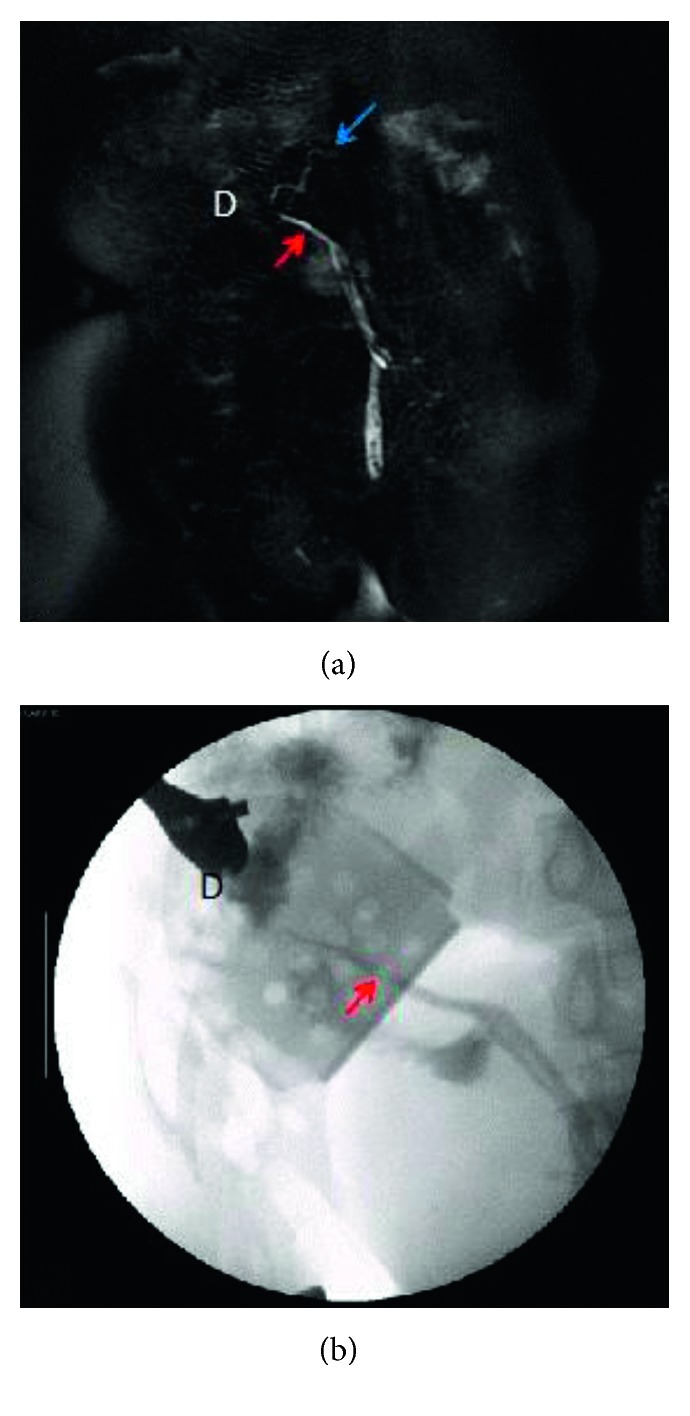
MRCP shown in (a) and intraoperative cholangiogram in (b) depicting the biliary tree with the gallbladder inferiorly. The hepatic ducts join to form the common hepatic and bile ducts (red arrow) that empty into the duodenum which has its usual right upper quadrant location. The pancreatic duct is depicted by the blue arrow. D = duodenum.

On physical examination, she was hemodynamically normal, and the only pertinent finding was tenderness to palpation in her midabdomen. All blood tests were within normal limits including a white blood cell count and liver function tests. Abdominal ultrasound demonstrated cholelithiasis and sludge in an intrahepatic gallbladder located in her ectopic liver, but no evidence of gallbladder wall thickening or pericholecystic fluid (Figure 1). Previous computed tomography (CT) scans demonstrated an ectopic liver located in the pelvis as well as an intrahepatic gallbladder and interval contracture of the gallbladder in subsequent scans (Figures 2 and 3). Magnetic resonance cholangiopancreatography (MRCP) demonstrated a relatively decompressed intrahepatic gallbladder in the pelvis joining the hepatic duct more superiorly with the common bile duct emptying into the duodenum, which has its usual right upper quadrant location (Figure 4). As the patient had been symptomatic for several months and her clinical status did not improve during a period of observation and trial of ursodiol, cholecystectomy was recommended. The patient was consented for open cholecystectomy due to her unusual anatomy and likely need for hepatotomy to access the gallbladder.

**Figure 5:**
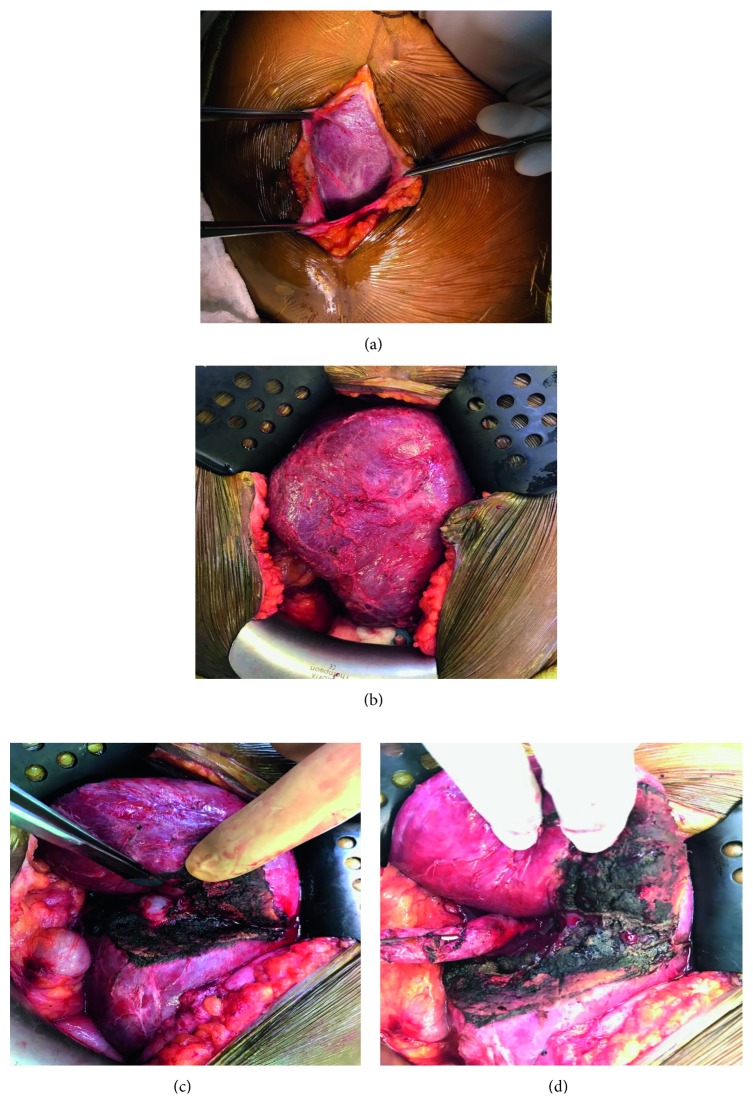
Midline laparotomy incision demonstrating the ectopic liver with adhesions (a, b). Hepatotomy exposing the intrahepatic gallbladder inferior to forceps (c) and dissected free of the surrounding parenchyma and retracted left laterally (d).

At laparotomy, the liver was encountered just beneath the fascia in the lower abdomen and was adhesed to the anterior abdominal wall and into the pelvis. A small portion of the gallbladder infundibulum was identified in a cleft on the underside of the liver once it was rotated right to left and cranially. The cleft was extended to the edge of the liver and down to the gallbladder with a recently introduced microwave ablation technology for hepatic parenchymal pretransection coagulation, to expose the gallbladder, which was then separated from the surrounding liver parenchyma (Figure 5). The cystic artery was identified, ligated, and divided. The cystic duct was identified, and a stone was found to be lodged in the cystic duct, which was then manipulated up into the fundus. The cystic duct was then ligated and divided, and a cholangiogram was performed via the cystic duct stump. The right and left hepatic ducts were visualized, as well as a long common bile duct, which traveled cranially to the right upper quadrant, where contrast was visualized within the duodenum (Figure 4). Ultrasound of the whole liver was then performed. There were no abnormal lesions noted. The patient recovered without major events and was discharged to home on postoperative day three. Final pathology revealed chronic cholecystitis with cholelithiasis.

## 3. Discussion

Intrahepatic gallbladder results from failure of the gallbladder to move from its intrahepatic position in the first trimester of gestation [9–12]. Intrahepatic gallbladders do not completely empty, leading to impaired function, contributing to stasis and cholelithiasis [13]. Patients with intrahepatic gallbladders, therefore, are susceptible to cholecystitis and other complications of cholelithiasis [14]. To our knowledge, this is the first report in the literature that describes a planned cholecystectomy for cholecystitis in an adult patient with an intrahepatic gallbladder in an ectopic pelvic liver. There are only two case reports in the literature of an intrahepatic gallbladder in a pelvic liver [8, 15]. Both of these patients had their aberrant anatomy discovered incidentally, and both had omphalocele repairs in infancy. Of note, in all three cases, the right and left hepatic duct joined to form the common bile duct that took a cranial course to enter the duodenal ampulla in the usual location. As patients after omphalocele repair are living longer, the existence of abnormal anatomy should be considered in these patients [16]. As care for patients with omphalocele improves, these patients are living longer. The existence of cholecystitis in pelvic intrahepatic gallbladders may become a more prevalent issue presented to surgeons. When evaluating these patients, planning MRCP in addition to intraoperative cholangiograms to study the “upside-down” biliary anatomy is a major benefit, allowing tracing and avoiding injury of the extrahepatic biliary tree.

In the case of the patient presented here, the use of ursodiol did not relieve her symptoms or cholecystitis. A review of literature has revealed varied efficacy of ursodiol in the nonsurgical treatment of chronic cholecystitis with return of symptoms in at least 50% of patients [17, 18]. Therefore, surgery remains the mainstay treatment for diseases of the gallbladder. Due to the rarity of this combination of intrahepatic gallbladder and ectopic liver, which causes an abnormal orientation of biliary anatomy, involving an experienced hepatobiliary surgeon is recommended.

Advances in surgical techniques and technology now allow preoperative recognition of aberrant anatomy and improved surgical planning. Multiple strategies are described and now used for parenchymal liver transection in hepatectomies, and while no method has been found to be superior, we elected to use a new approach for our hepatotomy in order to access the intrahepatic location of the gallbladder [19]. In recent years, microwave technology has been used with good results in pretransection coagulation of liver tissue prior to performing hepatotomy. This method, while not proven in large studies as superior to other methods, provides a fast and efficient way to hepatic parenchymal transection, minimizing blood loss and bile leaks [20–22].

There is no published data on risk of malignancy in pelvic livers. In this case, intraoperative ultrasound of the entire liver was completed to rule out any further abnormal anatomy or lesion. With no abnormal anatomy or lesion identified, no biopsy of the liver was taken.

## 4. Conclusion

As patients with a prior history of omphalocele are living longer, the existence of intrahepatic pelvic gallbladders is expected to be increasingly reported [16]. Gallbladder disease occurring in intrahepatic gallbladders poses an additional surgical risk to patients and adds to the complexity of the cholecystectomy procedure especially if the gallbladder is located in an ectopic pelvic liver. Obtaining preoperative planning MRCP and intraoperative cholangiogram is recommended. Expert hepatobiliary surgeon involvement is recommended as the combination of intrahepatic gallbladder and ectopic pelvic liver distorts the normal biliary and hepatic vascular anatomy. The use of microwave technology for pretransection coagulation of liver tissue during hepatectomy is a safe and effective method to employ during the above described procedures [20–22].
